# A survey of patient acceptability of the use of artificial intelligence in the diagnosis of paediatric fractures: an observational study

**DOI:** 10.1308/rcsann.2024.0008

**Published:** 2024-03-13

**Authors:** FHG Roberts, TRW Roberts, Y Gelfer, C Hing

**Affiliations:** ^1^ Epsom and St Helier Hospitals NHS Trust, UK; ^2^ St George’s University Hospitals NHS Foundation Trust, UK

**Keywords:** Artificial intelligence, Fracture, Paediatrics, Emergency, Orthopaedics

## Abstract

**Introduction:**

This study aimed to assess carer attitudes towards the use of artificial intelligence (AI) in management of fractures in paediatric patients. As fracture clinic services come under increasing pressure, innovative solutions are needed to combat rising demand. AI programs can be used to diagnosis fractures, but patient perceptions towards its use are uncertain.

**Methods:**

We conducted a cross-sectional survey of carers of paediatric patients presenting to fracture clinic at a tertiary care centre, combining single-best-answer questions and Likert-type questions. We investigated patient perception of clinical review in the emergency department (ED), disruption to school to attend fracture clinic, and attitudes towards AI.

**Results:**

Of the paediatric fracture patients participating in this study, 45% were seen within two hours, 29% were seen between two and four hours, and 26% were seen after four hours; 75% were seen by both a nurse and a doctor, 16% were seen only by a nurse and 9% only by a doctor. A total of 61% of children had to take time off school for their appointment and 59% of parents had to take time off. Of all respondents, 56% agreed that more research is needed to reduce waiting times, 76% preferred a nurse or doctor to review their child's radiograph, 64% were happy for an AI program to diagnose their child's fracture, and 82% were happy with an AI program being used as an adjunct to a clinician's diagnosis.

**Conclusions:**

Carer perceptions towards the use of AI in this setting are positive. However, they are not yet ready to relinquish human decision making to automated systems.

## Introduction

Musculoskeletal injuries in children account for nearly half of the four million presentations to Paediatric Emergency Departments (EDs) across the UK per year. Of these, fractures are an important cause of morbidity, with a reported incidence between 1,500 to 3,600 per 100,000 children per year.^1^ Most fractures do not require admission to hospital but may be managed as outpatients via local fracture clinics. The British Orthopaedic Association Standards for Trauma (BOAST) guidelines describe the standards of care that patients with a significant musculoskeletal injury should receive in an outpatient setting. The first point of guidance describes the timeframe for review by an Orthopaedic specialist, explaining that ‘patients should be seen in a new fracture clinic within 72-hours of presentation with the injury’.^2^ Timely assessment is essential to optimal management, with delays leading to increased pain and loss of opportunity, particularly in the paediatric population.^3^

Fracture clinic services throughout the UK have been under pressure in recent years and the mismatch between service demand and service availability continues to pose a challenge to orthopaedic specialists.^4^ The COVID-19 pandemic greatly exacerbated this problem, as an acute reduction in the provision of services and a shift in population health-seeking behaviour has compounded pressure on NHS services and increased patient backlogs.^5^ There is, therefore, an important and continued need for innovation in orthopaedics to help meet this demand, evolving outpatient orthopaedic services at pace with developing technologies.

Artificial intelligence (AI) has been defined as the ability of a computer to accomplish human-like tasks.^6^ In medicine, AI has been used as a diagnostic aide since the 1960s, where early-era devices provided statistical analyses of numerical data derived from radiological images to support human clinicians in their diagnoses.^7^ Advancements in both technological innovation and computer processing power have driven the development of increasingly complex and capable machines, with AI research now moving beyond simply mimicking intelligence and into the exploration of areas such as experiential learning.^6,7^

Today's AI has the potential to improve the diagnosis and management of myriad medical conditions and is already seeing effective use in specialities such as Oncology and Dermatology.^8,9^ In the orthopaedic setting, AI has seen a variety of applications, from clinical prognostication to outcome calculation. Notably, research has explored the use of AI in fracture identification with promising results. AI has been shown to perform at a level equal to human diagnosticians when diagnosing common fractures, and a specific AI outperformed both general physicians and orthopaedic surgeons in the setting of proximal humeral fracture diagnosis. AI has also been shown to equal human performance in recognising plain radiographic fractures of the ankle, wrist and hand with at least 83% accuracy. Yet evidence of the efficacy of AI in accurately diagnosing subtle and occult fractures is lacking.^10^

The relative novelty of AI in healthcare means there are many barriers to its successful implementation that are independent of the efficacy of the machine itself. Integration at an organisational level requires transparent collaboration between organisations and AI vendors, yet a paucity of vendors may render healthcare organisations vulnerable to acquiring inappropriate products, particularly where companies have a limited understanding of how to apply their AI to the individual needs of a healthcare organisation. There is also a wide range of computer literacy among clinicians and, although it is advantageous to develop user-friendly programs, this is not always possible. A highly effective AI may, therefore, be untenable if the clinicians it is directed at are unable to integrate it into their daily practice.^11,12^

Critically, AI must also be acceptable to patients. Little is yet known in this regard, particularly with respect to the paediatric population. The literature highlights the dehumanisation of the clinician – patient relationship, low trustworthiness of AI, and a perceived lack of regulation as key patient concerns, and, although patients may be comfortable with the use of AI as an adjunct in certain settings, they still exhibit a preference for a clinician.^13^ It is, therefore, essential to further elucidate patient opinion if AI is to be employed meaningfully in the future.

This study aimed to assess parent/carer attitudes towards use of AI in the management of orthopaedic injuries in paediatric patients.

## Methods

This study was a noninterventional, cross-sectional survey of parents or guardians of paediatric patients presenting to an outpatient orthopaedic fracture clinic at a tertiary care centre in London from June to August 2022. Parents or guardians of patients referred to the fracture clinic were invited to participate when checking into their appointment and before being seen by a clinician. Participation was voluntary and the study period was 12-weeks. The study was conducted as a service evaluation under audit guidelines and was registered with the trust audit department: registration number AUDI003065.

**Figure 1:**
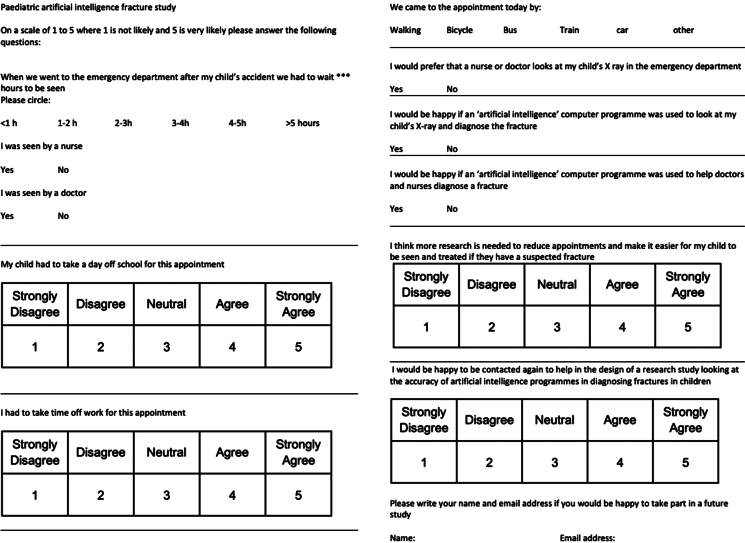
Questionnaire completed by parents of children attending the fracture clinic

The survey was an 11-item questionnaire (Figure 1). Data were collected on the child's initial presentation to the ED (length of time to be seen and whether they were seen by a doctor or nurse), disruption to school or work in order to attend the outpatient appointment and perceptions towards use of AI in managing orthopaedic injuries. Questionnaires were anonymous and no biometric or identifiable information was collected. Questions were either single best answer, or Likert-type with a scale ranging from strongly disagree to strongly agree.

Completed questionnaires were returned to a locked ‘post box’ held behind the reception desk. Responses were loaded manually onto a secure electronic database held on a Trust computer. Data were analysed using Microsoft Excel (Microsoft Corporation, version 16). Data were collected under the audit framework and thus ethical approval for this study was not required.

## Results

A total of 184 responses were obtained; 123 surveys were completed in full, with 61 surveys partially completed.

### Section 1 – Regarding the child's presentation to the ED

There were 141 complete responses to Section 1. Total waiting time to be seen by a clinician was represented in brackets of one hour, from less than one hour to more than five hours. Of Section 1 respondents, 24% (34/141) were seen within one hour, 21% (30/141) within two hours, 12% (17/141) within three hours and 13% (19/141) within four hours. A total of 13% (19/141) reported waiting longer than five hours. Of all Section 1 respondents, 75% (106/141) reported being seen by both a nurse and a doctor, 16% (22/141) were seen only by a nurse and 9% (12/141) were seen only by a doctor. One respondent reported not being seen by either.

### Section 2 – Disruption to work or school to attend an outpatient clinic

There were 165 complete responses to Section 2; 61% of respondents agreed (51/165) or strongly agreed (49/165) that their child had to take time off for the appointment, 14% (23/165) were neutral, and 25% either disagreed (19/165) or strongly disagreed (23/165). Of Section 2 respondents, 59% agreed (53/165) or strongly agreed (45/165) that they personally had had to take time off for the appointment, 11% (18/165) were neutral and 30% either disagreed (18/165) or strongly disagreed (31/165). A total of 56% of respondents agreed (52/165) or strongly agreed (40/165) that more research is needed to reduce waiting times, whereas 35% (58/165) were neutral, and 9% either disagreed (7/165) or strongly disagreed (8/165). There were 177 responses to the mode of transport used to attend fracture clinic appointment; 90% (159/177) of respondents attended their appointment at fracture clinic by either car, train or bus, and 69% (120/177) reported attending via private vehicle.

### Section 3 – Attitudes towards AI

There were 165 complete responses to Section 3, with 76% (125/165) of respondents saying they would prefer a nurse or doctor to review their child's radiograph. A total of 64% (105/165) said they would be happy if an AI program was used to diagnose their child's fracture, and 82% (135/165) reported being happy with an AI program being used to help in the diagnosis of fractures. A total of 8% (13/165) of respondents reported no preference for how their child's fracture was diagnosed but preferred AI not to be involved, 4% (7/165) reported no preference but would be happy for AI to assist in the diagnosis, and 12% (20/165) described no preference but would be happy for AI to make a diagnosis of fracture. Of the 165 respondents, 10% (16/165) would prefer a healthcare professional to make the diagnosis and preferred AI not to be involved, 15% (24/165) preferred a healthcare professional to make the diagnosis (and not AI) but would be open to having an AI programming assisting, and 52% (85/165) reported preferring a healthcare professional to make the diagnosis and being open to AI both assisting in, or making, the diagnosis of fracture.

## Discussion

Our results demonstrate a positive attitude towards the use of AI in diagnosing fractures in the paediatric setting. Only 18% of respondents did not want AI to assist in the diagnosis of their child's fracture and, whereas 76% preferred a healthcare professional to make the diagnosis of fracture, 82% were happy for AI to augment this interaction. A total of 16% had no preference for whether their child was seen by a clinician and would be happy for the process of fracture diagnosis to be automated. These results emulate data previously presented in this area,^14^ offering evidence in favour of automation of diagnoses in the paediatric setting, which has far-reaching implications.

The pathway of fracture management can be lengthy. Patients presenting to the ED are triaged, undergo an initial assessment, and then have imaging requested by a healthcare professional. Once this is reviewed and initial management suggested, most patients are then discharged home with an outpatient fracture clinic appointment for specialist orthopaedic review.^14^ Although well established, this process can be inefficient and is prone to bottlenecking. For instance, there is commonly a time-delay between initial assessment and subsequent suggested diagnosis in the ED. Poor staffing levels, high patient volume and/or acuity, and limited availability of services, in particular radiology reporting, have all been cited as possible influencing factors.^15,16^ The immediate-term consequences to patients include possible long waiting times and a delay in the acquisition of appropriate high-quality care.

Navigation through both the ED and fracture clinic can be very time intensive, causing significant disruptions to the patient and their carer. Regular and ongoing reviews at fracture clinic can compound this problem and may result in multiple missed days of school and work for both individuals. This survey's results support this idea; 55% of participants waited more than 2 hours to be seen in the ED and 23% waited more than four hours. Further, in the outpatient setting, 65% of participants agreed or strongly agreed that they had to take time off to attend the fracture clinic.

The negative effects of missing school on childhood academic attainment are well established. There is a proportionally detrimental effect of absence on attainment, with this effect beginning after just a few days' absence.^17^ As well as the important implications for patients highlighted above, this also incurs significant loss of departmental resources and clinician time. Reducing instances of absence and their duration is, therefore, highly important.

Innovations such as virtual fracture clinics, the use of which has increased significantly since the COVID-19 pandemic, have been effective at reducing the rate of referrals from the ED to fracture clinics and, consequently, school and work days missed.^18^ Interestingly, studies investigating the efficacy of virtual fracture clinics show that the rate of discharge, rather than onward face-to-face assessment, is between 33% and 60%,^19–21^ which implies that there are a significant number of unnecessary referrals made to the fracture clinic. Indeed, one centre found that 37% of paediatric fracture clinic referrals had no confirmed fracture before referral, and 29% of all suspected fractures were subsequently found not to have one.^22^ Using AI to improve diagnostic accuracy may serve to further reduce the rate of unnecessary referral, safeguard patient and carer time, and improve efficiency of hospital systems.

Although research into the development and implementation of AI in radiological diagnostics has existed for many years, the vast majority of current and historical AI programs represent investigational proofs of concept with minimal near-future clinical applications. A recent review examined the availability of licensed AI programs in this field, highlighting only six. Of these, 50% used plain radiography as their modality (OsteoDetect, FractureDetect, BoneView). Each demonstrates high sensitivity (88.0%–95.0%) and specificity (88.0%–90.2). One (OsteoDetect) shows a performance comparable with that of a clinician, and all have been shown to improve clinician performance when used as an augmentative measure.^23^ This is supported by a recent systematic review with meta-analysis, which found that, across all available literature, including grey literature, the “pooled diagnostic performance from the use of AI to detect fractures had a sensitivity of 92% and 91% and specificity of 91% and 91%, on internal and external validation, respectively”.^24^

However, no currently licensed AI program has been approved as the sole diagnostic agent capable of replacing a clinician, nor has any been licensed for use in the paediatric setting. Furthermore, current machine and deep-learning AI programs are designed to review specific body parts or regions, with no single program yet capable of performing at clinician level in all musculoskeletal regions. As such, they currently have limited practical application in isolation to one another, except in regions with high individual fracture prevalence.

The acceptability of AI to patients is a key factor that cannot be ignored. Yet, as highlighted earlier, research in this area is lacking, particularly in the paediatric population. One study, similar in design to this research, investigated the hypothetical use of an AI program versus a clinician in radiograph interpretation to explore patient perceptions of the use of AI as an adjunct to clinician diagnosis. It found significantly higher confidence of patients in the accuracy of a clinician's diagnosis when compared with AI (9.20 vs 7.06, *p*≤0.001) and, when asked to determine their preference in case of a disagreement between the two, 95.4% indicated a preference for a clinician. Additionally, this study reported a significantly higher patient confidence in AI-assisted interpretation versus AI-assisted management (7.06 vs 4.86, *p*≤0.001).^25^

Our study demonstrates a similar pattern, where the majority of carers reported a preference for a clinician (76%), but were open to AI being used as an adjunct to diagnosis (82%). Further research, particularly in the paediatric population, is needed to bolster these and other initial, promising results.

Utilising an AI program for autonomous fracture diagnosis may be beneficial at both the individual and departmental level. Obtaining a rapid diagnosis would allow for faster decision making and appropriate management strategies to be implemented, which could improve both patient safety and treatment outcomes by reducing waiting times and time to treatment. The varied skill mix and diagnostic confidence and accuracy of ED clinicians means that certain nonfracture injuries may be immobilised inappropriately and referred on for specialist review.

For individual clinicians, AI could improve the diagnostic accuracy and confidence of nonspecialists, thereby reducing cognitive load. This is significant as it reduces the risk of missed diagnoses that can result from cognitive fatigue.^26^ It may also give expert clinicians more time and mental capacity to review and diagnose more complex emergency pathologies. At the departmental level, implementing AI programs in imaging diagnostics has the potential to reconfigure patient streaming pathways, reducing bottlenecks to diagnosis and management, and reducing overall capacity issues through the ED.

Another positive impact of AI in this context may be through the reduction of unnecessary travel to outpatient appointments. In this survey, 69% of respondents used a private car to attend their appointment. The recent coronavirus pandemic has affected travel behaviours, with working-from-home becoming the norm for many, with fewer people preferring public transport or sustainable commuting in a private vehicle.^27^ Research has demonstrated that the rate of climate change is accelerating, which poses a threat to both the national and global public health gains of the last century.^28^ The Greener NHS Programme^29^ seeks to reduce the environmental impact of healthcare and create a sustainable model for the future.

Virtual fracture clinics, originally implemented to reduce the burden on outpatient services, already dovetail well with this initiative. They have been shown to be highly effective, improving patient outcomes and satisfaction, and reducing face-to-face attendances by up to half.^30^ AI would support this new green initiative, as a reduction in unnecessary appointments through improved diagnostic accuracy would reduce unnecessary vehicular travel.

There are several limitations that may negatively influence this study's results. The study's completion rate was only 67%. This may be due to the binary nature of several of the questions, as people may not have felt the answers available were representative of their opinions. Similarly, the questionnaire was divided into three sections that covered different time periods during their child's management journey, meaning that some respondents may have been unable to remember or recall information accurately. The data collection period was also relatively short, and no biometric data were collected, which limits generalisability. The wording of the questionnaire may also be limiting, as respondents were not asked directly if they would prefer an AI program over a human to diagnose a fracture in their child. Expanding the questionnaire to obtain a more detailed understanding of respondent preferences would serve to significantly strengthen these initial results.

## Conclusion

This study assessed participant attitudes towards the use of AI in the diagnosis of fractures in the paediatric setting. The results show that perceptions towards the use of AI in this context are positive, but that carers still prefer a clinician with respect to fracture diagnosis. Patient education around AI and its potential benefits may improve its acceptability as a diagnostic tool.
